# Influence of Impurities on the Front Velocity of Sputter Deposited Al/CuO Thermite Multilayers

**DOI:** 10.3390/ma14237224

**Published:** 2021-11-26

**Authors:** Altangerel Dulmaa, Diederik Depla

**Affiliations:** Department of Solid State Sciences, Ghent University, Krijgslaan 281 (S1), 9000 Gent, Belgium; Dulmaa.Altangerel@ugent.be

**Keywords:** thermite, multilayer, magnetron sputter deposition, impurities

## Abstract

CuO and Al thin films were successively deposited using direct current (reactive) magnetron sputter deposition. A multilayer of five bilayers was deposited on glass, which can be ignited by heating a Ti resistive thin film. The velocity of the reaction front which propagates along the multilayer was optically determined using a high-speed camera. During the deposition of the aluminum layers, air was intentionally leaked into the vacuum chamber to introduce impurities in the film. Depositions at different impurity/metal flux ratios were performed. The front velocity reaches a value of approximately 20 m/s at low flux ratios but drops to approximately 7 m/s at flux ratios between 0.6 and 1. The drop is rather abrupt as the front velocity stays constant above flux ratios larger than 1. This behavior is explained based on the hindrance of the oxygen transport from the oxidizer (CuO) to the fuel (Al).

## 1. Introduction

For many applications, energy storage and harvesting should be down-scaled to the nano- and microscale [1,2]. Thin-film batteries and reactive multilayers are examples of chemical energy storage approaches in this field [3]. Reactive multilayers are composed of alternating layers of two materials, which are involved in an oxidation/reduction reaction or in a compound formation reaction. The multilayer is in a metastable state, with excess chemical energy [4]. When the multilayer is locally activated by an external energy source, the two materials mix or react, transform to a stable state, and release heat. The heat is partially transferred to the non-reacted part of the sample, which can result in a self-sustained system where the reaction propagates through the sample without additional external input [5].

Thermites are mixtures of a metal (the fuel), and a metal oxide (the oxidizer) which are in the given context present as thin films in a multilayer design. When ignited, the thermite undergoes an exothermic oxidation-reduction reaction involving the transfer of oxygen from the oxidizer to the fuel. Thermite reactions have been known since the 19th century, when Goldsmidth and Vautin described the reaction of a mixture of aluminum and iron oxide powder that produces aluminum oxide and metallic iron [6]. Bulk thermite powders have been used in many applications such as the repair of faulty heavy machinery castings, rail welding, forgings, stern frames of ships and large pump housing. As multilayers, thermites have been used as miniature pyrotechnical ignition devices to trigger airbag inflation, arm and fire devices, and micro-propulsion systems [7]. The majority of published studies have focused on compositions that use aluminum as fuel. Typical oxidizers are MoO_3_, CuO, and Fe_2_O_3_; however, tenorite (CuO), and Al multilayers are leading materials due to their high thermal output, which is approximately 4 kJ/g [3,4,8].

Many parameters, such as film thickness, multilayer design, and substrate choice, have been studied to optimize the aluminum/tenorite multilayer system [9]. Nicollet et al. [10] have investigated the effect of the heating surface area on the ignition characteristics. The ignition time can be changed from 0.5 s to 60 μs by decreasing the heating surface area or more generally by increasing the power density to 8000 W·cm^−2^. The minimum ignition time at constant surface area and power depends on the multilayer design. Reducing the number of bilayers from 15 to 5 by increasing layer thickness, while keeping the total thickness constant, results in an increase of the ignition time from 283 to 556 μs. The substrate choice is important because substrates with high thermal conductivity act as a heat sink, and considerably affect the self-sustainability of the reaction. Thus, ignition and sustained combustion cannot be obtained when the multilayers are deposited on highly conductive substrates [11,12,13]. The burning rate or the reaction front velocity can be tuned through a change of the bilayer thickness. Larger front velocities are obtained [10,13,14,15] for decreasing bilayer thicknesses. The composition of the multilayer also greatly influences the front velocity, as the reactant ratio directly impacts the overall film thermal conductivity [13,14]. As an example, the front velocity is reduced when the Al/CuO reactant ratio increases. As Al is much more conductive than CuO, enriching the multilayer with aluminum induces an important increase in the multilayer global conductivity, leading to a decrease in the front velocity. During the deposition, an intermixing naturally occurs between the metal and oxide layers to form a diffusion barrier that can affect the reaction kinetics [14].

In this work, we investigate the role of intentionally added impurities during the deposition of the aluminum layers, which act as fuel in the sputter-deposited Al/CuO thermite multilayers. The motivation for this work is based on the discrepancy between the base pressures obtained in laboratory and industrial set-ups. Experiments performed on the lab scale are often performed in state-of-the-art (ultra-) high-vacuum systems with base pressures lower than $1\times 10^{-3}$ Pa, whereas industrial (mainly large area) systems often work at base pressures, starting from this boundary, but are generally even higher. As in both systems, the deposition rate is similar (at least for magnetron sputtering), the impurity content will be different. When the film properties depend strongly on the impurity content, this could hinder to transfer of the lab-obtained results to industry. For example, Zapata et al. [16] report on the front velocity of identical multilayers deposited in two different experimental set-ups. Samples from one deposition system burn systematically at higher front velocity. Although many experimental parameters influence the final properties of the multilayers, such as film stress, thermal conductivity, and interface mixing, it is remarkable that the two set-ups have a different base pressure. The films produced in the more pure chamber ($1\times 10^{-5}$ Pa) systematically burn (between 1.3 and 4.5 times) slower than the ones produced in the other chamber ($5\times 10^{-5}$ Pa). Comparison of the influence of the base pressure is difficult, however, because no deposition rates have been reported, and hence it is not possible to calculate the impurity-to-metal flux ratio. A second motivation to study the influence of impurities is that during the deposition of the multilayers, the deposition conditions are switched between a pure argon discharge (aluminum) and an oxygen/argon discharge (CuO). A too-short interval between these alternating deposition conditions could affect the properties of the aluminum layer as the remaining oxygen gas could act as an impurity. The aforementioned interval is not reported in some studies on thermite reactions.

## 2. Experimental

The experiments were performed in a high-vacuum stainless steel cylindrical chamber (30 cm in diameter and 70 cm in length) pumped with a combination of a turbomolecular and rotary pumps. The base pressure, as measured with a Penning gauge, was lower than 4 × 10^−4^ Pa. Two magnetrons were used to deposit the CuO and Al thin films. The angle between both 2 inch planar magnetrons was 90${}^{\circ }$. Metallic Al and Cu targets (99.999%, HMW Hauner GmbH & Co. KG, Bayern, Germany) were mounted on the magnetrons. The magnetrons were powered with a DC power supply (Hüttinger 1500 DC). The substrate was mounted at a rotatable holder, which permitted the substrate to be placed parallel to one of the two magnetrons. The perpendicular distance between target and substrate was 10 cm. During the deposition of one material, a shutter protected the target of the other magnetron. In between two consecutive depositions, the substrate was retracted from the deposition zone, and the deposition of the following layer was prepared by opening/closing the shutters, sputter-cleaning the target, and controlling the gas atmosphere. This processing interval lasted long enough to ensure the pumping of the oxygen and air from the system after the deposition of CuO and the deposition of Al, respectively. The argon and oxygen flows were controlled by two mass flow controllers (MKS Instruments, Rochester, NY, USA). The oxygen and argon flow rates were set at 39 and 73 standard cubic centimeters per minute, respectively. The pressure during deposition was measured with a capacitance gauge (Pfeiffer Vacuum, Berliner, Germany). The oxygen fraction was calculated as the ratio of the oxygen partial pressure and the total pressure. Table 1 summarizes the experimental conditions. The deposition conditions of CuO were based on previous work to optimize the film properties of the copper oxide thin films [17]. The samples were not intentionally heated nor cooled.

**Figure 1:**
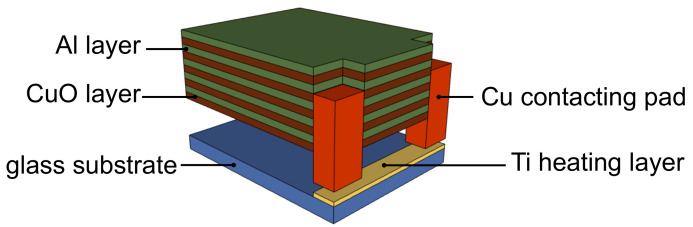
Schematic overview of the built-up sample used to study the thermite reaction (not to scale). The multilayer and the pads in the drawing are lifted to expose the Ti heating layer. Prior to the deposition of the multilayer, a titanium layer was deposited on the edge of the substrate. Contacting electrical pads, made from Cu tape, were placed on top of the titanium layer. The Cu pads were shielded during the deposition of the multilayers. Five bilayers were deposited. The first layer deposited was a CuO thin film.

Prior to the multilayer depositions, a ≈400 nm thick titanium layer (Table 1) was sputter-deposited onto the edge of the cleaned glass substrate, where it served as a Joule heater to ignite the thermite reaction. Copper tape was used for the contacting electrical pads. Afterwards, the substrate with the Ti film with the shielded Cu pads was introduced into the vacuum chamber to deposit alternating CuO and Al layers to form the multilayer stack. A schematic overview of the built-up sample is shown in Figure 1. Based on the work of Bahrami et al. [14] the same film thickness for both Al and CuO was chosen. According to this latter study, this choice is optimal for a complete reaction.

To study the influence of impurities on the reaction front velocity, the ratio between the impurity flux and the aluminum metal flux was varied. Air was leaked into the vacuum chamber during the deposition of aluminum using a needle valve. The impurity flux was calculated from the base pressure prior to the deposition, as discussed in [18,19]. The aluminum metal flux was calculated based on the film thickness and the film density. The film thickness was measured using a contact profilometry approach (Taylor-Hobson, Leicester, UK)), whereas the film density was determined by means of X-ray reflectometry (Bruker, Ettlingen, Germany)).

The electrical resistivity of the multilayer was measured with a four-probe resistivity set-up. The resistivity was measured over the sample for at least five different positions. The relative error was not larger than 2%. To check the reproducibility of the measurements, experiments were repeated at four different τ values (0.27, 0.46, 1.53, and 4.41). The average relative error on the measured resistivity was approximately 20%.

X-ray diffraction (XRD) patterns were measured to determine the domain size of the aluminum thin film and the aluminum lattice parameter embedded in the multilayer. The domain size was calculated from the full width at half maximum (FWHM) using the Scherrer equation. Peak fitting of the (111) Bragg reflection using a pseudo-Voigt profile showed only a Lorentzian contribution excluding the microstrain as a broadening mechanism.

**Figure 2:**
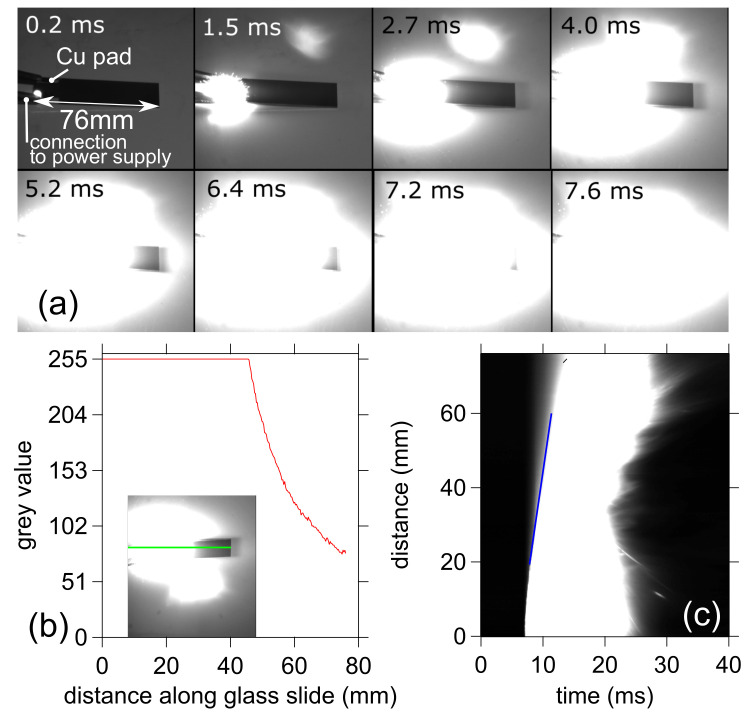
(**a**) Snapshots taken during multilayer reaction front velocity measurement using the ultra-high speed camera FASTCAM SA4. The dark region is the non-reacted part of the sample. (**b**) Measured line profile. The inset shows the image, together with the profile line (green). (**c**) Compilation of line profiles to determine the front velocity. The blue line indicates the boundary between the dark and white region used to determine the velocity. The slope of the blue line corresponds to the average velocity (mm/ms).

Self-sustained reactions are characterized by a propagating reaction wavefront that moves through the energetic multilayer. The propagation velocity or reaction front velocity has been measured by tracking light emission related to the reaction front, assuming a one-dimensional propagation [20,21]. An ultra-high-speed camera (FASTCAM SA4, Photron Europe Limited, High Wycombe, UK)) was placed in front of the sample, approximately half a meter away to record the reaction. The framing rate was set as 10,000 frames per second. The frame-by-frame tracking with the Phantom camera software was used to extract the data (Figure 2a).

The reaction can easily be detected as the boundary between the region with strong light emission (white) and the dark region (black). Per image, the data along a line profile placed along the long edge of the substrate observed (Figure 2b) was extracted using the image analysis package embedded in Igor Pro (WaveMetrics, Lake Oswego, OR, USA). The data in each line profile were compiled in a two-dimensional matrix, as shown in Figure 2c. The front velocity was determined at different moments by fitting a line at the boundary between the dark and white region. The average of 10 measurements in the steady region was used to determine the average velocity and its variance.

## 3. Results

### 3.1. XRD Analysis

**Figure 3:**
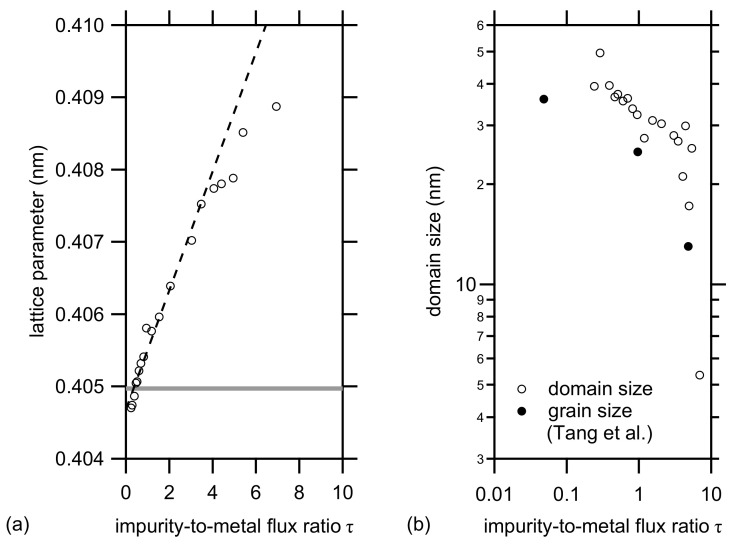
Lattice parameter (**a**) and domain size (**b**) of the aluminum thin films embedded in the multilayer as a function of the impurity-to-metal flux ratio τ. The lattice parameter was determined based on the position of the (111) Bragg reflection. The domain size was calculated from the FWHM of the same peak. The full markers refer to the grain size determined by TEM for aluminum layers deposited at different base pressures as published by Tang and co-workers [23].

The position of the Al (111) XRD Bragg reflection shifts towards lower 2θ values for increasing impurity-to-metal flux ratios, $\tau =F_{impurity}/F_{metal}$. The lattice parameter of Al was determined based on the position of the (111) peak. Except for the highest values for τ, a linear increase was observed as a function of τ. At low values of τ, the lattice parameter is slightly lower than the excepted value for pure aluminum [22] (Figure 3a). As the lattice parameter is determined only on one peak, it is difficult to draw any conclusions from this difference.

As discussed in [18,19], the influence of the embedded impurities on the microstrain is negligible. Hence, based on curve-fitting of the same peak using a Lorentzian profile, the FWHM was determined and the domain size was calculated using the Scherrer equation. The domain size gets smaller at higher impurity levels. Except for the highest values, a power law behavior is observed. The obtained values can be compared with the values published by Tang et al. [23]. In the latter paper, the effect of embedded impurities on the mechanical properties of sputter-deposited aluminum layers is investigated. The grain size was determined by transmission electron microscopy. As discussed by Dulmaa et al. [18], a good agreement between the grain size measured with TEM and the domain size measured with XRD is found for small grains. The data of Tang et al. are in the same range as our value. The calculation of τ for this case will be discussed further in the paper.

### 3.2. Front Velocity Measurements

**Figure 4:**
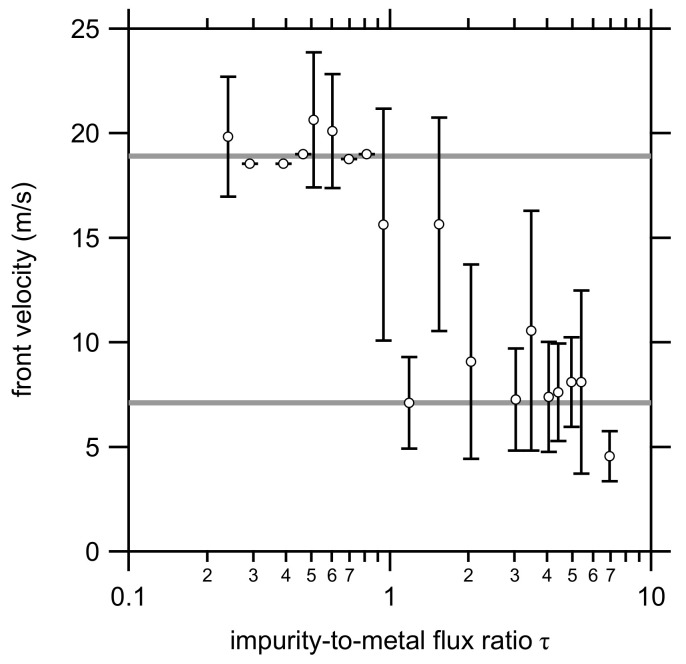
The front velocity of the thermite reaction as a function of the impurity-to-metal flux ratio τ. The two gray lines indicate the weighted average of the two observed groups.

The front velocity of the deposited multilayers was determined based on the method described in Section 2 (Figure 2). In contrast to the lattice parameter and the domain size, no gradual change in the front velocity was observed. Two groups can be identified (Figure 4), i.e., a group with a high front velocity (on average 18.9 m/s) at τ values lower than approximately 1, and a group with a low front velocity (on average 7.1 m/s) at higher τ values.

### 3.3. Thermal Conductivity

The electrical resistivity of the multilayer was measured using a four-probe resistivity set-up. It is important to remark, as depicted in Figure 1, that the final layer of the stack is an aluminum layer. The electrical resistivity of bulk CuO (≈2.5 Ω·m [24]) is several orders of magnitude higher than the bulk resistivity of Al (2.650 × 10^−8^ Ω·m [25]). Hence, it can safely be assumed that the electrical resistivity of the multilayer is defined by the film properties of Al because the resistivity of the multilayer can be approximated by a parallel circuit model [26]. This statement probably also holds for the films deposited in the most impure conditions (τ≈7.6) as the maximum measured resistivity of the multilayer was 1.850 × 10^−8^ Ω·m. The obtained resistivity values were converted into thermal conductivities via the application of the Wiedemann–Franz law. This is an approximation because it is known that the Wiedemann–Franz or the Lorenz ratio can be a function of the domain size [27]. The ratio between grain size and the electron mean free path drives this latter dependency. As the electron mean free path for aluminum is 18.9 nm [28], the effect can be estimated as small because for film deposited at large values of τ the domain size is still of the order of the mean free path. The good agreement between frequency-domain thermoreflectance measurements and calculated values based on the Wiedemann–Franz law, as reported by Schmidt et al. [29], confirms this reasoning.

**Figure 5:**
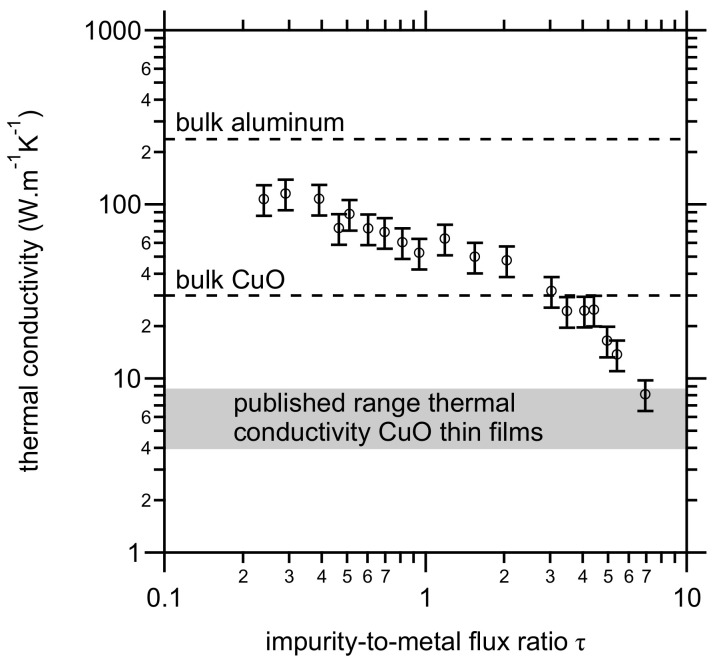
Thermal conductivity of aluminum as a function of the impurity-to-metal flux ratio τ. The thermal conductivity was calculated based on the Wiedemann–Franz law from four-probe resistivity measurements. The indicated range is based on the data by Hartung et al. [42] and Kusiak et al. [38].

The obtained result of our calculation is shown in Figure 5. The thermal conductivity decreases from approximately 100 W·m^−1^·K^−1^ to 10 W·m^−1^·K^−1^ with increasing impurity-to-metal flux ratio τ. Similar values as for the purest films have been reported by Boiko et al. [30] for thermal evaporated and sputter-deposited thin films [29]. Values close to the bulk value have been reported by Lugo and Oliva [31]. For the sake of further discussion, the obtained values are compared with the thermal conductivity of both Al and CuO. The thermal conductivity of bulk Al equals to 237 W·K^−1^·m^−1^ [25]. This value is approximately double the thermal conductivity of the purest thin films i.e., deposited at low τ values. Values of the thermal conductivity of bulk CuO are scarce. Two values for bulk samples can be found in different publications. A value of 17 W·K^−1^·m^−1^ is reported in several papers [32,33,34,35,36]. This value is only referenced to Gmelin [37] in the work by Kusiak et al. [38]. However, in this compilation on the properties of CuO, the actual value of thermal conductivity at room temperature is not mentioned. The work contains a reference to the measurements by Ntifiez Regueiro et al. [39], who report a value of ≈30 W·K^−1^, and not 17 W·K^−1^·m^−1^. The value of ≈30 W·K^−1^·m^−1^ is also reported by Liu et al. [40] without reference. A more recent measurement [41] confirms the measurement by Ntifiez Regueiro et al. Moreover, Kusiak et al. have measured the thermal conductivity of sputter-deposited CuO thin films and report a value of ≈ 4 W·K^−1^·m^−1^. Hartung et al. measured approx. 8.6 W·K^−1^·m^−1^ likewise for sputter-deposited thin films [42]. Hence, in the same fashion as for the presented Al measurements, the latter two studies show a similar drop in thermal conductivity when compared to the corresponding bulk value.

## 4. Discussion

The dependence of flame speed or front velocity *v* on different parameters has been described by Armstrong [43], who suggested the following equation,

(1) $$v^{2}=\frac{3R}{\delta^{2}}\frac{T_{f}^{2}\lambda^{2}}{E\left(T_{f}-T_{o}\right)}A\exp\left(-\frac{E}{RT_{f}}\right)$$

In the following section, the different parameters will be discussed and confronted with the experimental results.

In Equation (1), *R* is the gas constant. δ represents 1/4 of the bilayer thickness. The inverse relationship between the bilayer thickness and the front velocity has been confirmed by several researchers [4]. In the experiments presented here, the value of the bilayer thickness remained unchanged and can therefore be treated as a constant.

**Figure 6:**
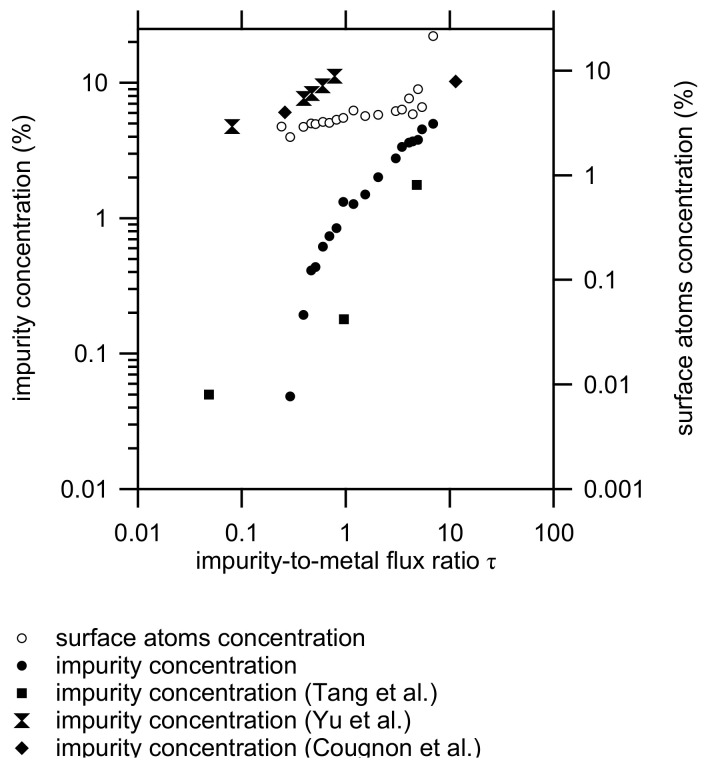
Calculated impurity concentration (closed markers) and the concentration of surface atoms (open markers) as a function of the impurity-to-metal flux ratio τ. Literature values are also presented based on the work of Tang et al. [23], Yu et al. [46] and Cougnon et al. [47].

The flame temperature $T_{f}$ influences the front velocity in several ways. First, the mass diffusivity will increase at higher temperatures and is described by the Arrhenius-like part of Equation (1) (see below). Reported values for the flame temperature (and adiabatic reaction temperatures) of the Al/CuO system are often larger than 2000 K [8,12,13,16] i.e., large in comparison to room temperature $T_{0}$. Hence, Equation (1) can be simplified, and except for the Arrhenius-like part, the front velocity shows a $T_{f}^{1/2}$ dependence. Although experimental studies show that the flame temperature can be larger than the adiabatic reaction temperature of the Al/CuO system, this temperature can be used as a first approximation, as is often performed in modeling [44]. The adiabatic reaction temperature is defined by the material properties and the reaction chemistry. In our experiments, the films are deposited under identical conditions, except for the addition of small quantities of air during the deposition of the aluminum layers. As such, it can be expected that the chemical properties of the deposited layers will not change dramatically. Nevertheless, it would be interesting to obtain compositional information on the deposited material. However, the detection of low quantities of impurities in the embedded aluminum layers is not straightforward. An estimate can be made based on the observed lattice expansion (Figure 3). If it is assumed that the lattice expansion is due to the insertion of impurities into octahedral interstitial voids of the face-centered cubic lattice of aluminum, the concentration of impurities $C_{imp}$ can be calculated as

(2) $$C_{imp}=\frac{a-a_{0}}{r_{imp}-\left(\sqrt{2}-1\right)r_{M}}$$

where $a_{0}$ is the lattice parameter for pure aluminum, $r_{imp}$ is the radius of the impurity atom, and $r_{M}$ is the metallic aluminum radius. The ionic radius for oxygen and nitrogen is similar, and has an average radius of 0.143 nm. The radius of the metal atoms can be calculated from the lattice parameter as 0.143 nm. In the calculation, we assume that the minor difference between the pure aluminum lattice parameter and the first measurement is negligible. The calculated impurity concentration is presented in Figure 6 (with closed round markers).

To test the validity of this approach, the result can be confronted with literature data. Tang et al. [23] have measured the oxygen concentration of aluminum film deposited at different base pressures using atom probe tomography (APT). The work of Tang et al. mentions no deposition rates, but based on earlier results by the same team, a deposition rate of 0.3 nm/s is estimated [45]. The impurity flux was calculated based on the reported base pressure. As the experiments were performed in pulse mode, the impurity flux was doubled to account for the exposure to the impurities during the off-time of the experiment. As shown in Figure 6, similar concentrations are obtained. Much higher oxygen levels were reported by Yu and Thompson [46] during the e-beam evaporation of nickel thin films. Similar results were obtained by Cougnon et al. [47] for sputter-deposited NiCr. This behavior is understandable, as the initial sticking coefficient of oxygen on nickel is on the order of 0.8 [48], whereas for aluminum, values between 0.005 [49] and 0.107 [50] have been reported i.e., one to two orders of magnitude lower as compared to Ni (or NiCr), or stated differently, recalculating the incorporation probability brings the concentration within the same range as the presented results. In summary, although it is a rough calculation, the obtained result seems reasonable. The low concentration of impurities (<1%) confirms the initial idea that they can have little effect on the chemistry of the reaction. Therefore, it can be concluded that the abrupt change in the reaction front velocity is probably not due to a change in the flame temperature.

In Equation (1), λ represents the thermal diffusivity, which can be calculated from the thermal conductivity, the heat capacity, and the material density. The film density for the Al thin films was measured as a function of τ and, as reported previously [19], no systematic changes were observed. Models use different approaches to calculate the thermal conductivity of a multilayer and show that the method has little impact on the final results [51] which is in agreement with the work of Armstrong [43]. As the thermal conductivity of the aluminum layers remain up to the highest value of τ larger than the reported values for CuO thin films (Figure 5), the change in the thermal conductivity seems not to be critical in explaining the drop in the front velocity as a function of τ. Finally, the thermal diffusivity depends also on the specific heat capacity. As mentioned before, from a chemical point of view, the influence should be small because the chemical composition of the deposited films hardly change. However, as shown by [52], the heat capacity of thin films depends also on the grain (domain) size, but the effect is rather small. Furthermore, conductive and radiative heat losses play a role in determining the characteristics of the reaction. The effects of radiative heat losses on reaction velocities are generally quite small and can be ignored if reaction velocities are high [5]. Conductive heat losses typically have a more significant influence on the front velocity [53]. As demonstrated by Rossi [9], a strong heat loss due to a thermal conductive substrate hinders the propagation of the reaction. However, the substrate was not changed in our study.

The last parameters in Equation (1) describe the mass diffusivity, i.e., *A* and *E*, the Arrhenius pre-factor, and the activation energy for mass diffusion, respectively. Simulations show that the rate-limiting step during the reaction of Al/CuO thin films is the transport of oxygen towards the aluminum [13,44], where the formation of an aluminum oxide interfacial layer can play a critical role [10,14]. Here, we suppose a similar mechanism. As shown in previous work [19,47], the grain refinement can be explained by impurities which act as nucleation centers during the film growth. Hence, the impurities are embedded in the grains, as demonstrated by atom probe tomography. Based on the measured XRD domain size, it is possible to calculate the fraction of surface atoms per domain size [54]. As shown in Figure 6, this idea is consistent with the fact that the fraction of surface atoms for the grains is indeed much larger than the impurity concentration, or stated differently, the grain surface cannot be completely covered by the impurity atoms. Due to the generated heat, the film temperature will increase, which makes the oxygen atoms mobile. They will diffuse, due to their low solubility in aluminum, towards the grain boundaries. Indeed, as demonstrated by Tang et al. [23], oxygen atoms reside preferentially in the grain boundaries. The sample preparation for APT in the latter study was performed at room temperature. In contrast to low-temperature sample preparation [55], this could influence the measurement of the grain boundary concentration due to Ga ion implantation. Nevertheless, the results illustrate the high mobility of oxygen in aluminum towards the grain boundaries. Simultaneously with the transport towards the grain boundaries, the high temperature will induce grain growth. The latter was, for example, demonstrated by Zweiacker in pulsed laser heating experiments of aluminum thin films [56]. At distances close to the melting pool (approximately 30 μm), a strong increase in the average grain diameter is observed, with a change in the grain size distribution. Due to the grain growth, the number of surface atoms will decrease, and it can be expected that at a given impurity-to-metal flux ratio, the oxygen layer will cover the grains. A similar reasoning was followed by Iyer and Wong [57]. These authors explained the observed grain configuration in annealed aluminum thin films based on a critical oxygen concentration in the grain boundary, which slows down the grain boundary movement and/or the coalescence of adjacent grains. The formation of an oxide layer, also known as a “tissue” layer, on the surface of aluminum grains was also demonstrated by Barna and Adamik [58] and was used to explain the observed changes in thin film growth as a function of the oxygen content in the deposited aluminum films [59]. As mentioned before, the transport of oxygen through the alumina layer is expected to be the rate-limiting step, and hence it can be expected that at a given impurity-to-metal flux ratio, the tissue layer formation is completed, which results in a strong decrease in the front velocity.

In the paragraphs above, we mainly focused on the possible mechanisms related to the parameters in Equation (1). The proposed reason i.e., the formation of a diffusion barrier for oxygen, is a possible reason which needs further investigation. Indeed, due to the complexity of the thermite multilayer reaction, other mechanisms could also influence the front velocity. For example, in the work of Zapata et al. [16], the film stress was indicated as a possible reason for the difference in behavior between two series of experiments. Highly stressed films could prematurely delaminate, and this behavior can influence the measured front velocity. A difference of approximately 30 MPa was observed, and identified as a possible reason for the discrepancy between both series of experiments. As shown by Yu et al. [46], the embedded impurities will lead to a compression stress in the film. Based on the strain measured using XRD, it is possible to calculate the film stress according the equations provided by Yu et al. [46]. A value of approximately −500 MPa (compressive stress) was obtained for the most impure thin film, whereas for a τ value of approximately 1 i.e., at the drop of the front velocity, the compressive stress was approximately −100 MPa. Although these values do not exclude a critical behavior, it is remarkable that the difference between pure and impure film is much larger than the difference reported by Zapata et al. Moreover, one should realize that even at temperatures below the melting temperature of aluminum, the thermal stresses in the multilayer are quite large. At an ignition temperature close to the melting temperature of aluminum, e.g., 500 ${}^{\circ }$C, the thermal stress of a pure aluminum thin film deposited at glass is approximately −625 MPa. However, thin film delamination is only observed after several thermal cycles [60]. The calculated values seem to exclude a stress-based mechanism to explain the drop in the front velocity at a given impurity-to-metal flux ratio. As the thermal expansion coefficient of CuO [61] is 11.5 × 10^−6^/K, i.e., approximately half of the value for aluminum [25], similar values will be obtained for CuO.

## 5. Conclusions

Aluminum layers, which form together with CuO thin films a thermite multilayer, were contaminated with impurities leaked into the sputtering chamber during the depositions. The influence of the impurity-to-metal flux ratio on the reaction front velocity was studied. It was shown that the front velocity dramatically decreases at a critical flux ratio. The role of different parameters which can influence the front velocity was critically investigated. Based on our analysis, we suggest that the formation of an aluminum oxide tissue layer covering the aluminum grains hinders the transport of oxygen from the CuO oxidizer to the aluminum fuel. Further study is required to validate the proposed mechanism, but the study shows that good control of the vacuum conditions during the production of thermite multilayers is required to obtain consistent results. The embedded impurities can, on the other hand, also be used to control the reaction dynamics of the thermite.

## Figures and Tables

**Table 1:** Deposition conditions of the different used materials i.e., the titanium contact layer, and the Al and CuO layers to form the bilayer. The oxygen fraction is calculated as the ratio of the oxygen partial pressure and the total pressure.

Parameter	Ti	Al	CuO
Target material	Ti	Al	Cu
Argon pressure $P_{Ar}$ (Pa)	0.8	0.5	0.5
Oxygen fraction $f_{O_{2}}$	0	0	0.28
Discharge current $I_{d}$ (A)	0.7	0.5	0.3
Target-to-substrate distance *T*-*S* (cm)	7	10	10
Layer thickness (nm)	400	100	100
Deposition rate (nm/s)	0.91	0.83	1.25

## Data Availability

The data presented in this study are available on request from the corresponding author.

## References

[1] Mariello M., Guido F., Mastronardi V.M., Madaro F., Mehdipour I., Todaro M.T., Rizzi F., Vittorio M.D. (2021). Micro- and nanodevices for wind energy harvesting. Nano Tools and Devices for Enhanced Renewable Energy.

[2] Silva J.P.B., Sekhar K.C., Pan H., MacManus-Driscoll J.L., Pereira M. (2021). Advances in Dielectric Thin Films for Energy Storage Applications, Revealing the Promise of Group IV Binary Oxides. ACS Energy Lett..

[3] Rossi C., Zhang K., Esteve D., Alphonse P., Tailhades P., Vahlas C. (2007). Nanoenergetic materials for MEMS: A review. J. Microelectromech. Syst..

[4] Adams D.P. (2015). Reactive multilayers fabricated by vapor deposition: A critical review. Thin Solid Films.

[5] Weihs T.P. (2014). Fabrication and characterization of reactive multilayer films and foils. Metallic Films for Electronic, Optical and Magnetic Applications.

[6] Goldschmidt H., Vautin C. (1898). Aluminium as a heating and reducing agent. J. Soc. Chem. Ind..

[7] Salvagnac L., Assie-Souleille S., Rossi C. (2020). Layered Al/CuO thin films for tunable ignition and actuations. Nanomaterials.

[8] Fischer S.H., Grubelich M.C. (1998). Theoretical Energy Release of Thermites, Intermetallics, and Combustible Metals.

[9] Rossi C. (2018). Engineering of Al/CuO reactive multilayer thin films for tunable initiation and actuation. Propellants Explos. Pyrotech..

[10] Nicollet A., Lahiner G., Belisario A., Souleille S., Djafari-Rouhani M., Estève A., Rossi C. (2017). Investigation of Al/CuO multilayered thermite ignition. J. Appl. Phys..

[11] Manesh N.A., Basu S., Kumar R. (2010). Experimental flame speed in multi-layered nano-energetic materials. Combust. Flame.

[12] Amini-Manesh N., Basu S., Kumar R. (2011). Modeling of a reacting nanofilm on a composite substrate. Energy.

[13] Lahiner G., Nicollet A., Zapata J., Marín L., Richard N., Rouhani M.D., Rossi C., Estève A. (2017). A diffusion–reaction scheme for modeling ignition and self-propagating reactions in Al/CuO multilayered thin films. J. Appl. Phys..

[14] Bahrami M., Taton G., Conédéra V., Salvagnac L., Tenailleau C., Alphonse P., Rossi C. (2014). Magnetron sputtered Al-CuO nanolaminates: Effect of stoichiometry and layers thickness on energy release and burning rate. Propellants Explos. Pyrotech..

[15] Petrantoni M., Rossi C., Salvagnac L., Conédéra V., Estève A., Tenailleau C., Alphonse P., Chabal Y.J. (2010). Multilayered Al/CuO thermite formation by reactive magnetron sputtering: Nano versus micro. J. Appl. Phys..

[16] Zapata J., Nicollet A., Julien B., Lahiner G., Esteve A., Rossi C. (2019). Self-propagating combustion of sputter-deposited Al/CuO nanolaminates. Combust. Flame.

[17] Dulmaa A., Vrielinck H., Khelifi S., Depla D. (2019). Sputter deposition of copper oxide films. Appl. Surf. Sci..

[18] Dulmaa A., Cougnon F.G., Dedoncker R., Depla D. (2021). On the grain size-thickness correlation for thin films. Acta Mater..

[19] Cougnon F.G., Dulmaa A., Dedoncker R., Galbadrakh R., Depla D. (2018). Impurity dominated thin film growth. Appl. Phys. Lett..

[20] Weismiller M., Malchi J., Yetter R., Foley T. (2009). Dependence of flame propagation on pressure and pressurizing gas for an Al/CuO nanoscale thermite. Proc. Combust. Inst..

[21] Sullivan K.T., Kuntz J.D., Gash A.E. (2014). The Role of Fuel Particle Size on Flame Propagation Velocity in Thermites with a Nanoscale Oxidizer. Propellants Explos. Pyrotech..

[22] Nakashima P.N.H. (2019). The crystallography of aluminum and its alloys. Encyclopedia of Aluminum and Its Alloys.

[23] Tang F., Gianola D.S., Moody M.P., Hemker K.J., Cairney J.M. (2012). Observations of grain boundary impurities in nanocrystalline Al and their influence on microstructural stability and mechanical behaviour. Acta Mater..

[24] Linnera J., Sansone G., Maschio L., Karttunen A.J. (2018). Thermoelectric properties of p-type Cu_2_O, CuO, and NiO from hybrid density functional theory. J. Phys. Chem. C.

[25] Lide R.L. (2005). CRC Handbook of Chemistry and Physics, Internet Version 2005.

[26] Chen Y.Y., Juang J.Y. (2016). Finite element analysis and equivalent parallel-resistance model for conductive multilayer thin films. Meas. Sci. Technol..

[27] Wei-Gang M., Wang H.D., Xing Z., Koji T. (2009). Different effects of grain boundary scattering on charge and heat transport in polycrystalline platinum and gold nanofilms. Chin. Phys. B.

[28] Gall D. (2016). Electron mean free path in elemental metals. J. Appl. Phys..

[29] Schmidt A.J., Cheaito R., Chiesa M. (2010). Characterization of thin metal films via frequency-domain thermoreflectance. J. Appl. Phys..

[30] Boiko B.T., Pugachev A.T., Bratsychin V.M. (1973). Method for the determination of the thermophysical properties of evaporated thin films. Thin Solid Films.

[31] Lugo J.M., Oliva A.I. (2016). Thermal properties of metallic films at room conditions by the heating slope. J. Thermophys. Heat Transf..

[32] Abolghasemi M., Keshavarz A., Mehrabian M.A. (2012). Thermodynamic analysis of a thermal storage unit under the influence of nano-particles added to the phase change material and/or the working fluid. Heat Mass Transf..

[33] Ahmed M., Yusoff M., Ng K., Shuaib N. (2014). Effect of corrugation profile on the thermal–hydraulic performance of corrugated channels using CuO–water nanofluid. Case Stud. Therm. Eng..

[34] Kim H., Ham J., Park C., Cho H. (2016). Theoretical investigation of the efficiency of a U-tube solar collector using various nanofluids. Energy.

[35] Jegadheeswaran S., Sundaramahalingam A., Pohekar S.D. (2019). High-conductivity nanomaterials for enhancing thermal performance of latent heat thermal energy storage systems. J. Therm. Anal. Calorim..

[36] Desmukh K.B., Karmare S.V. (2021). A review on convective heat augmentation techniques in solar thermal collector using nanofluid. J. Therm. Eng..

[37] Gmelin E. (1992). Cupric oxide-CuO: Its structural, electrical, thermal and magnetic properties. Indian J. Pure Appl. Phys..

[38] Kusiak A., Battaglia J.L., Gomez S., Manaud J.P., Lepetitcorps Y. (2006). CuO thin films thermal conductivity and interfacial thermal resistance estimation. Eur. Phys. J. Appl. Phys..

[39] Ntifiez Regueiro M., Castello D., Jaime M. (1991). Thermal conducitivity of high temperature superconductors. Physica B.

[40] Liu M., Lin M.C., Wang C. (2011). Enhancements of thermal conductivities with Cu, CuO, and carbon nanotube nanofluids and application of MWNT/water nanofluid on a water chiller system. Nanoscale Res. Lett..

[41] Yoshida N., Naito T., Fujishiro H. (2013). Thermoelectric Properties of Li-Doped CuO. Jpn. J. Appl. Phys..

[42] Hartung D., Gather F., Hering P., Kandzia C., Reppin D., Polity A., Meyer B.K., Klar P.J. (2015). Assessing the thermoelectric properties of CuxO (x = 1 to 2) thin films as a function of composition. Appl. Phys. Lett..

[43] Armstrong R. (1990). Models for gasless combustion in layered materials and random media. Combust. Sci. Technol..

[44] Tichtchenko E., Estève A., Rossi C. (2021). Modeling the self-propagation reaction in heterogeneous and dense media: Application to Al/CuO thermite. Combust. Flame.

[45] Gianola D.S., Van Petegem S., Legros M., Brandstetter S., Van Swygenhoven H., Hemker K.J. (2006). Stress-assisted discontinuous grain growth and its effect on the deformation behavior of nanocrystalline aluminum thin films. Acta Mater..

[46] Yu H.Z., Thompson C.V. (2015). Stress engineering using low oxygen background pressures during Volmer-Weber growth of polycrystalline nickel films. J. Vac. Sci. Technol. A Vac. Surf. Films.

[47] Cougnon F.G., Schramm I.C., Depla D. (2019). On the electrical properties of sputter deposited thin films: The role of energy and impurity flux. Thin Solid Films.

[48] Winkler A., Rendulic K.D., Wendl K. (1983). Quantitative measurement of the sticking coefficient for oxygen on nickel. Appl. Surf. Sci..

[49] Brune H., Wintterlin J., Trost J., Ertl G., Wiechers J., Behm R.J. (1993). Interaction of oxygen with Al(111) studied by scanning tunneling microscopy. J. Chem. Phys..

[50] Leroy W.P., Mahieu S., Persoons R., Depla D. (2009). Method to determine the sticking coefficient of O_2_ on deposited Al during reactive magnetron sputtering using mass spectrometry. Plasma Process. Polym..

[51] Alawieh L., Knio O.M., Weihs T.P. (2011). Effect of thermal properties on self-propagating fronts in reactive nanolaminates. J. Appl. Phys..

[52] Yu J., Tang Z., Zhang F., Ding H., Huang Z. (2009). Measurement of the heat capacity of copper thin films using a micropulse calorimeter. J. Heat Transf..

[53] Jayaraman S., Mann A.B., Reiss M., Weihs T.P., Knio O.M. (2001). Numerical study of the effect of heat losses on self-propagating reactions in multilayer foils. Combust. Flame.

[54] Agrawal D.C. (2013). Introduction to Nanoscience and Nanomaterials.

[55] Lilensten L., B. Gault B. (2020). New approach for FIB-preparation of atom probe specimens for aluminum alloys. PLoS ONE.

[56] Zweiacker K. (2015). In-situ TEM Investigation of Rapid Solidification of Aluminum and Aluminum Copper Alloys. Ph.D. Thesis.

[57] Iyer S.S., Wong C.Y. (1985). Grain growth study in aluminum films and electromigration implications. J. Appl. Phys..

[58] Barna P.B., Adamik M. (1998). Fundamental structure forming phenomena of polycrystalline films and the structure zone models. Thin Solid Films.

[59] Petrov I., Barna P.B., Hultman L., Greene J.E. (2003). Microstructural evolution during film growth. J. Vac. Sci. Technol. A Vac. Surf. Films.

[60] Koike J., S. Utsunomiya S., Y. Shimoyama Y., K. Maruyama K., Oikawa H. (1998). Thermal cycling fatigue and deformation mechanism in aluminum alloy thin films on silicon. J. Mater. Res..

[61] Lumpp J.K., Chen N., Goretta K.C. (1990). Mechanical properties of CuO. High Temp. Mater. Process..

